# ﻿Three new species of the genus *Tetragnatha* Latreille, 1804 (Araneae, Tetragnathidae) from China

**DOI:** 10.3897/zookeys.1125.86905

**Published:** 2022-10-20

**Authors:** Song-lin Li, Ping Liu, Xian-jin Peng

**Affiliations:** 1 College of Life Sciences, Hunan Normal University, Changsha, Hunan 410081, China Hunan Normal University Changsha China

**Keywords:** Araneoidea, long-jawed spider, taxonomy, Tetragnathinae

## Abstract

Three new species of *Tetragnatha* Latreille, 1804 are described from China: *T.bifurcata* Li & Liu, **sp. nov.** (♂♀) and *T.tortilis* Li & Liu, **sp. nov.** (♂♀) from Yunnan Province, and *T.bimaculata* Li & Liu, **sp. nov.** (♂♀) from Hubei and Hunan provinces. Detailed descriptions, photographs of somatic features and copulatory organs, and a distribution map of these three species are provided.

## ﻿Introduction

*Tetragnatha* Latreille, 1804 is the largest genus of the family Tetragnathidae, currently comprising 322 species distributed worldwide, of which 51 species are known from China (Li and Lin 2016; WSC 2022). The Chinese species of *Tetragnatha* are relatively well studied by Zhu et al. (2003), who reviewed the Chinese fauna comprising 36 species. Since 2003, an additional seven species have been reported from this country (Zhao and Peng 2010; Barrion et al. 2011, 2013). However, approximately half of the species recorded or described from China are only known from a single sex (Li and Lin 2016).

While examining specimens collected from the Gaoligong and Wuling mountains, three new species of *Tetragnatha* were recognized and are described here.

## ﻿Materials and methods

Specimens were collected by beating shrubs and hand picking, and were stored in 75% ethanol. The epigyne were cleaned with trypsin solution before examination and photography. Left male palps and chelicerae were used for description and photography. Specimens were examined and measured with a Leica M205C stereomicroscope. Photographs were taken with a Kuy Nice E3IS PM digital camera mounted on an Olympus BX53 compound microscope and focus-stacked images were generated using Helicon Focus v. 7.6.1 and then modified in Adobe Photoshop CS2. The map was created by the online mapping software SimpleMappr (Shorthouse 2010). All measurements are given in millimeters (mm). Leg measurements are given in the following order: total length (femur, patella + tibia, metatarsus, tarsus). All specimens are deposited in the College of Life Sciences, Hunan Normal University, Changsha City, China (**HNU**). The terminology follows Castanheira and Baptista (2020).

### ﻿Abbreviations used in the text and figures

**Eyes**:

**ALE** anterior lateral eye;

**AME** anterior median eye;

**AME–AME** distance between AME;

**AME–ALE** distance between AME and ALE;

**PLE** posterior lateral eye;

**PME** posterior median eye;

**PME–PME** distance between PME;

**PME–PLE** distance between PME and PLE;

**MO** median ocular quadrangle.

**Chelicera**:

**AXl** auxiliary guide tooth of the lower row of chelicera;

**AXu** auxiliary guide tooth of the upper row of chelicera, above Gu;

**Ds** dorsal spur of chelicera;

**Gl** guide tooth of the lower row of chelicera;

**Gu** guide tooth of the upper row of chelicera;

**L2–n** teeth on the lower row of chelicera numbered from the distal end after Gl;

**OC** outer cusp;

**rsu** remaining proximal teeth on the upper row of male chelicera after ‘T’;

**sl** first major tooth after Gu in the upper row of male chelicera;

**T** elongated tooth in the upper row of male chelicera;

**U2–n** teeth on the upper row of chelicera numbered from the distal end after Gu.

**Palps and epigyne**:

**C** conductor;

**E** embolus;

**F** fold;

**K** knob;

**P** paracymbium;

**Sp** spermatheca;

**TL** translucent lobe;

**Y** cymbium.

## ﻿Taxonomic account

### ﻿Family Tetragnathidae Menge, 1866

#### ﻿Genus Tetragnatha

Taxon classificationAnimaliaAraneaeTetragnathidae

Latreille, 1804

149E8151-C37D-57A6-9093-1F981AFD379F

##### Type species.

*Araneaextensa* Linnaeus, 1758 from Sweden.

#### ﻿ Tetragnatha bifurcata

Taxon classificationAnimaliaAraneaeTetragnathidae

Li & Liu sp. nov.

A5144B72-36BB-59A8-892A-8CC41D591751

https://zoobank.org/27CEAEB3-4814-4DFD-AAB4-745CE7C240A1

Figs 1 , 2 , 7

##### Type material.

***Holotype*** ♂: China, Yunnan Province: Tengchong County, Houqiao Township, Zhaobitang Village, 25.5378°N, 98.2094°E, 2480 m, 29.V.2006, X.P. Wang & P. Hu leg. (Wang060529-1). ***Paratypes***: 2♂♂ 3♀♀, same data as holotype (Wang060529-1); 5♂♂, Tengchong County, Houqiao Township, Zhaobitang Village, 25.3986°N, 98.3053°E, 2374 m, 27.V.2006, X.P. Wang & P. Hu leg. (Wang060527-2); 1♂ 1♀, Lushui County, Luzhang Township, Yaojiaping River 25.9772°N, 98.7109°E, 2527 m, 19.V.2005, D. Kavanaugh et al. leg. (2005-015A).

##### Etymology.

The specific epithet is derived from the Latin adjective *bifurcus*, referring to the bifurcate distal end of the conductor.

##### Diagnosis.

**Figure 1.**
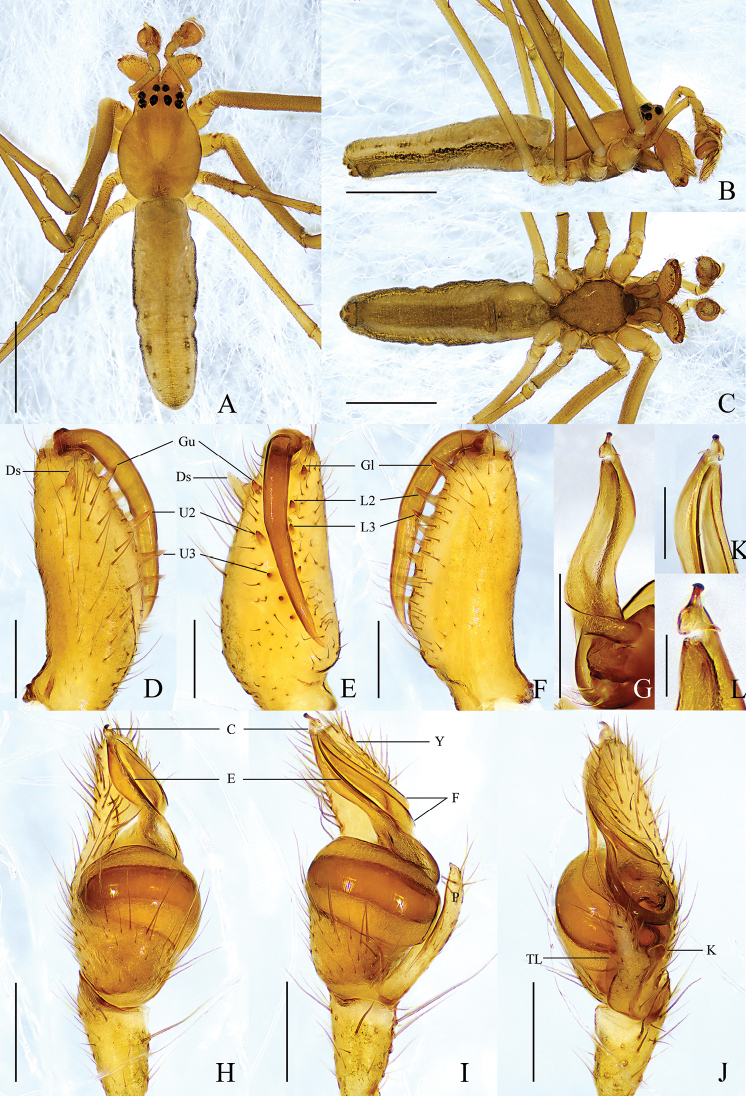
*Tetragnathabifurcata* sp. nov., holotype ♂ **A–C** habitus **A** dorsal view **B** lateral view **C** ventral view **D–F** left chelicera **D** upper view **E** inner view **F** lower view **G, K, L** conductor and embolus detail **G** dorsal view **H–J** left palp **H** prolateral view **I** ventral view **J** retrolateral view. Scale bars: 1 mm (**A–C**); 0.5 mm (**D–F**); 0.1 mm (**G**); 0.2 mm (**H–J**); 0.05 mm (**K, L**).

**Figure 2.**
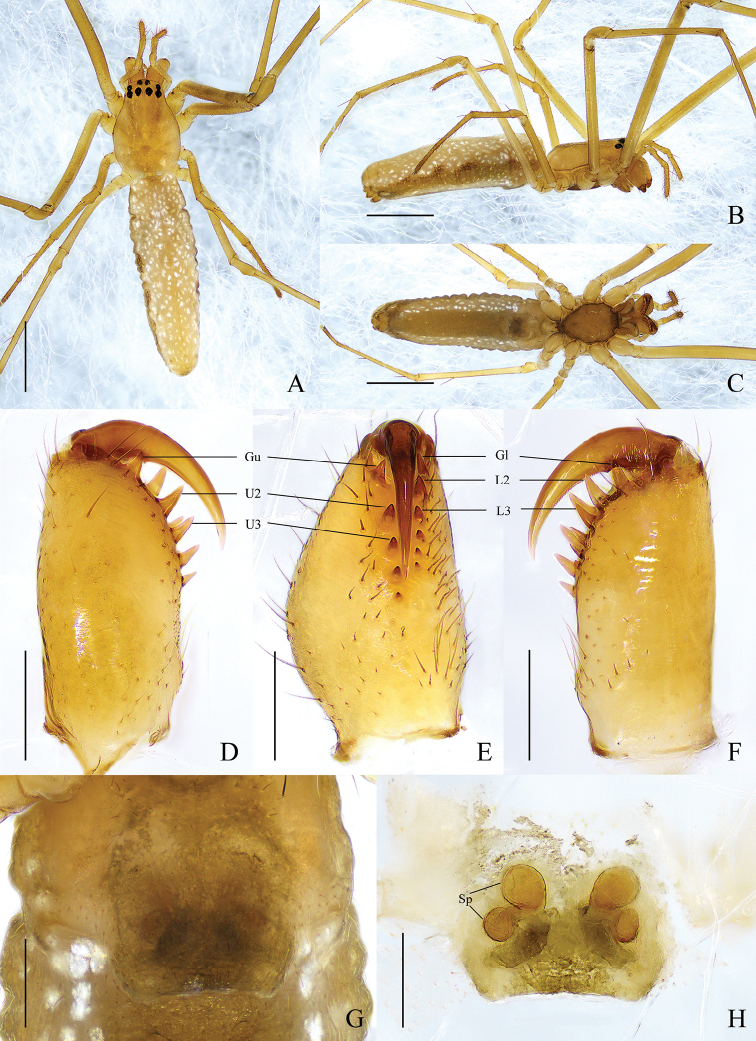
*Tetragnathabifurcata* sp. nov., paratype ♀ **A–C** habitus **A** dorsal view **B** lateral view **C** ventral view **D–F** left chelicera **D** upper view **E** inner view **F** lower view **G, H** female genitalia **G** epigynal fold, ventral view **H** vulva, dorsal view. Scale bars: 1 mm (**A–C**); 0.5 mm (**D–F**); 0.2 mm (**G, H**).

The new species resembles *T.tortilis* sp. nov. (Figs 5, 6). Males of the two species are similar in having a tapered dorsal spur on the chelicera, and the conductor with 2 folds, but can be distinguished by: (1) the distal portion of conductor bifurcated in *T.bifurcata* sp. nov. (Fig. 1L) (vs. not bifurcated; Fig. 5K); (2) the paracymbium with a pointed tip and terminal part located beyond the tegulum in ventral view (Fig. 1I) (vs. with blunt tip and terminal part located at the middle part of the tegulum; Fig. 5I). Females of the two species are similar in the shape of the epigynal fold and the absence of a central membranous sac in the vulva, but can be distinguished by: (1) the distance between the guide tooth and the second tooth of the upper row of chelicera slightly longer than the distance between the second tooth and the third tooth of the upper row of chelicera in *T.bifurcata* sp. nov. (Fig. 2E) (vs. the distance between the guide and the second tooth of the upper row of chelicera 3× longer than the distance between the second tooth and the third tooth of the upper row of chelicera; Fig. 6E); (2) the spherical anterior spermathecae are ~ 1.5× larger than the posterior spermathecae (Fig. 2H) (vs. anterior spermathecae oval and ~ 5× larger than the posterior spermathecae; Fig. 6H).

##### Description.

**Male** (holotype) (Fig. 1A–F). Total length 3.60. Carapace 1.31 long, 0.92 wide, yellowish brown, fovea and cervical and radial grooves distinct. Eye sizes and interdistances: AME 0.06, ALE 0.12, PME 0.12, PLE 0.10; AME–AME 0.08, AME–ALE 0.12, PME–PME 0.12, PME–PLE 0.10. MO anterior width 0.17, posterior width 0.24, length 0.22. Clypeus 0.08 high. Labium dark brown, with thickened edge. Sternum brown with dark edge. Legs yellowish brown, with sparse spines. Leg measurements: I, 11.45 (3.12, 3.84, 3.23, 1.26); II, 8.15 (2.38, 2.72, 2.19, 0.86); III, 3.73 (1.32, 1.04, 0.91, 0.46); IV, 7.16 (2.42, 2.21, 1.86, 0.67). Chelicera: ~ 1/2 carapace length; dorsal spur tapered, with blunt tip; *AXu* absent; upper row with 5 teeth: *Gu* slightly smaller than *U2*, *U2* almost equal to *U3* in size, distance between *U2* and *Gu* longer than distance between *U2* and *U3*, other teeth decreasing in size gradually; *AXl* absent; lower row with 5 teeth: *Gl* slightly smaller than *L2*, *L2* largest, other teeth decreasing in size gradually. Abdomen 2.26 long, 0.72 wide, dorsum yellowish brown, with 5 pairs of dark spots laterally and brown longitudinal line medially, both lateral sides with longitudinal dark band throughout entire abdomen; venter yellowish brown, median band brown.

Palp (Fig. 1G–L). Paracymbium with pointed tip, notch shallow, translucent lobe elongated, ~ 1/3 width of paracymbium, knob thumb-shaped. Tegulum oval, ~ 2× as wide as long. Conductor with 2 folds, distal portion bifurcated, upper branch thicker with slightly swollen tip, lower branch thinner with blunt tip. Embolus partly enveloped by conductor.

**Female** (Wang060529-1) (Fig. 2A–F). Total length 4.02. Carapace 1.38 long, 0.91 wide. Eye sizes and interdistances: AME 0.06, ALE 0.06, PME 0.07, PLE 0.07; AME–AME 0.05, AME–ALE 0.10, PME–PME 0.09, PME–PLE 0.09. MO anterior width 0.17, posterior width 0.24, length 0.20. Clypeus 0.04 high. Leg measurements: I, 10.31 (2.85, 3.50, 2.92, 1.04); II, 7.13 (2.13, 2.30, 1.92, 0.78); III, 3.24 (1.09, 0.94, 0.80, 0.41); IV, 6.39 (2.20, 1.98, 1.63, 0.58). Chelicera: *AXu* absent; upper row with 5 teeth: *Gu* slightly smaller than *U2*, *U2* largest, other teeth almost equidistant and decreasing in size gradually; *AXl* absent; lower row with 6 teeth: *Gl* slightly smaller than *L2*, *L2* largest, other teeth decreasing in size gradually. Abdomen 2.71 long, 0.85 wide, dorsum without dark spots but with dispersed pale spots; venter with pale spots on both sides. Color paler than that in male.

Epigyne (Fig. 2G, H). Fold ~ 3× wider than long. Vulva composed of 2 pairs of spherical spermathecae, diameter of anterior pair ~ 1.5× of posterior pair, anterior pair spaced by 1 diameter of anterior spermatheca, posterior pair spaced by 3 diameters of posterior spermatheca. Central membranous sac absent.

##### Distribution.

Known only from the type locality (Fig. 7).

#### ﻿ Tetragnatha bimaculata

Taxon classificationAnimaliaAraneaeTetragnathidae

Li & Liu sp. nov.

21429C10-3329-50BA-AE1F-0580B0A89ADE

https://zoobank.org/928B67AD-F5F6-47BC-8DEB-CD5B30C282B0

Figs 3 , 4 , 7

##### Type material.

***Holotype*** ♂: China: Hubei Province, Xuanen County: Shadaogou Township, Yuquan River, 29.7114°N, 109.7278°E, 805 m, 1.V.2016, W. Liu et al. leg. (HNU-HB-IV-1610). ***Paratypes***: 4♂♂ 4♀♀, same data as holotype (HNU-HB-IV-1610); 1♂, Sidaoshui Village, 29.6846°N, 109.5791°E, 602 m, 2.V.2016 (HNU-HB-IV-1611), 6♂♂, Wanzhai Township, Dongping Dam, 30.1470°N, 109.6127°E, 519 m, 4.V.2016 (HNU-HB-IV-1613), W. Liu et al. leg. Hunan Province, Shimen County, Huping Township: 2♀♀, Quanping Village, Zhipeng River, 30.0131°N, 110.5980°E, 611 m, 15.VI.2014 (HPS140615), 2♀♀, Jinbanshan Village, Yanshan Road, 30.0066°N, 110.5653°E, 520 m, 13.VI.2014 (HPS140613), 1♀, Quanping Village, 30.0123°N, 110.5432°E, 935 m, 18.VI.2014 (HPS140618), J.H. Gan et al. leg.

##### Etymology.

The specific epithet is the combination of the prefix *bi*- (two) and the Latin adjective *maculatus* (with spot), referring to the two dark spots on the posterior part of the abdomen.

##### Diagnosis.

**Figure 3.**
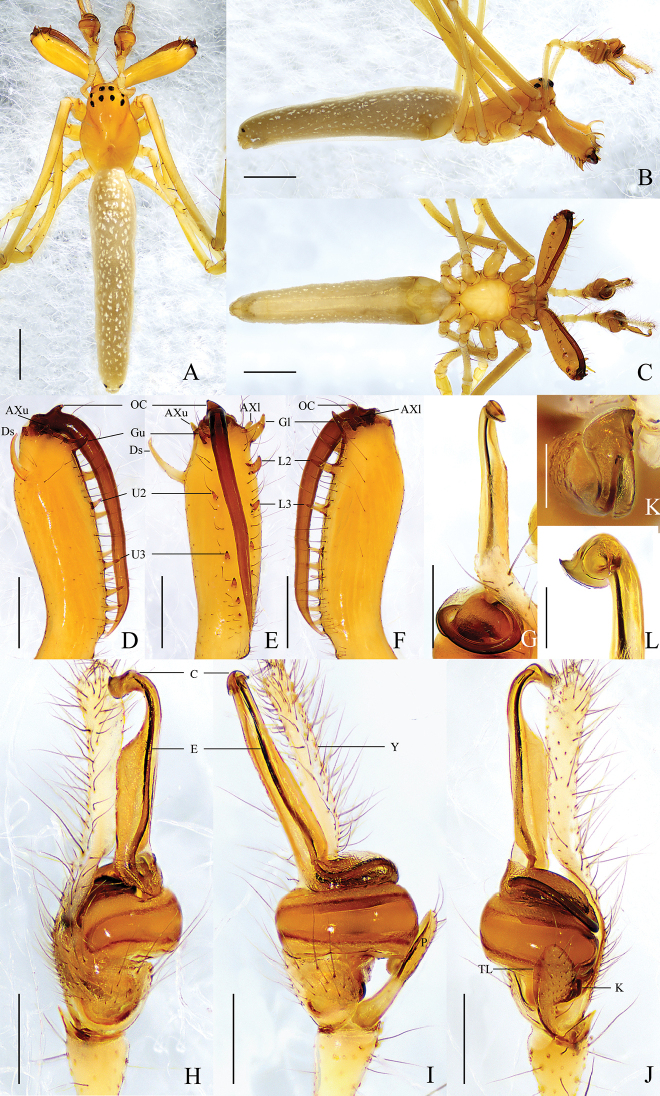
*Tetragnathabimaculata* sp. nov., holotype ♂ **A–C** habitus **A** dorsal view **B** lateral view **C** ventral view **D–F** left chelicera **D** upper view **E** inner view **F** lower view **G, K, L** conductor and embolus detail **H–J** left palp **H** prolateral view **I** ventral view **J** retrolateral view. Scale bars: 1 mm (**A–C**); 0.3 mm (**D–J**); 0. 1 mm (**K, L**).

**Figure 4.**
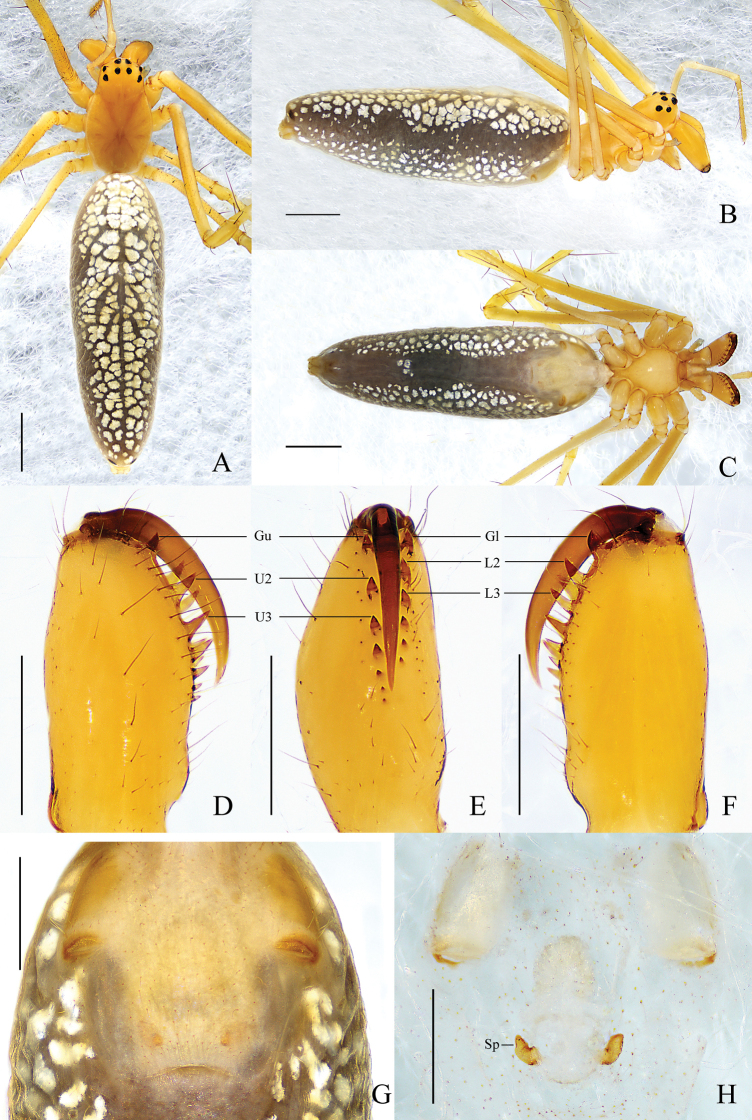
*Tetragnathabimaculata* sp. nov., paratype ♀ **A–C** habitus **A** dorsal view **B** lateral view **C** ventral view **D–F** left chelicera **D** upper view **E** inner view **F** lower view **G, H** female genitalia **G** epigynal fold, ventral view **H** vulva, dorsal view. Scale bars: 1 mm (**A–C**); 0.5 mm (**D–H**).

The males of this new species resemble those of *T.tanigawai* Okuma, 1988 (Okuma 1988: fig. 3A–G) in the elongate and curved dorsal spur of chelicera, the fang with an outer cusp, and the expanded proximal part of the conductor, but can be distinguished by: (1) the distance between the guide tooth and the second tooth of the upper row of chelicera almost equal to the distance between the second and third teeth of the upper row of chelicera in males of *T.bimaculata* sp. nov. (Fig. 3D) (vs. the distance between the guide tooth and the second tooth of the upper row of chelicera > 3× longer than the distance between the second and third teeth of the upper row of chelicera; fig. 3A in Okuma [1988]); (2) the conductor is ~ 3× longer than tegulum (Fig. 3I) with proximal 2/3 expanded (Fig. 3H) (vs. the conductor is ~ 2× longer than the tegulum (fig. 3G in Okuma [1988]) with proximal 1/2 part expanded; fig. 3D in Okuma [1988]); (3) the distal portion of conductor is almost rounded (Fig. 3G, H) (vs. with notch; fig. 3E, G in Okuma [1988]). The females of this new species resemble that of *T.esakii* Okuma, 1988 (Zhu et al. 2003: fig. 62A–F) in the shape of epigynal fold, the presence of a pair of spermathecae, and the absence of a central membranous sac in the vulva, but can be distinguished by the spermathecae that are bean-shaped and ~ 2× longer than wide in *T.bimaculata* sp. nov. (Fig. 4H) (vs. almost claviform and ~ 4× longer than wide; fig. 62F in Zhu et al. [2003]).

##### Description.

**Male** (holotype) (Fig. 3A–F). Total length 5.85. Carapace 1.59 long, 1.20 wide, yellow, fovea and cervical and radial grooves distinct. Eye sizes and interdistances: AME 0.11, ALE 0.08, PME 0.08, PLE 0.09; AME–AME 0.10, AME–ALE 0.17, PME–PME 0.15, PME–PLE 0.15. MO anterior width 0.28, posterior width 0.30, length 0.29. Clypeus 0.16 high. Labium yellow. Sternum pale yellow. Legs yellow, with sparse spines. Leg measurements: I, 20.04 (5.24, 6.43, 6.56, 1.81); II, 14.25 (4.16, 4.69, 4.24, 1.16); III, 7.53 (2.70, 2.21, 1.99, 0.63); IV, 12.23 (4.18, 3.80, 3.49, 0.76). Chelicera: almost as long as carapace; dorsal spur elongated, with pointed and curved tip; *AXu* present, with blunt tip; upper row with 7 teeth: *Gu* largest, distance between *U2* and *Gu* almost equal to distance between *U2* and *U3*, *U2*–*U4* almost equal in size, other teeth decreasing in size gradually; *AXl* present, thumb-shaped; lower row with 5 teeth: *Gl* largest, with widened base, other teeth gradually decreasing in size; fang with an outer cusp at base. Abdomen 4.27 long, 0.94 wide, dorsum pale yellow with serried pale spots and single pair of dark spots posteriorly; venter pale yellow, with paler spots on both sides.

Palp (Fig. 3G–L). Paracymbium with blunt tip, notch inconspicuous, translucent lobe elongate, ~ 1/6 of width of paracymbium, knob with a truncated tip. Tegulum oval, ~ 2× wider than long. Conductor without fold, proximal 2/3 of conductor expanded dorsally, distal portion twisted and curved towards dorsal side. Embolus completely enveloped by conductor.

**Female** (paratype HNU-HB-IV-1610) (Fig. 4A–F). Total length 6.82. Carapace 1.75 long, 1.16 wide. Eye sizes and interdistances: AME 0.10, ALE 0.08, PME 0.09, PLE 0.10; AME–AME 0.12, AME–ALE 0.16, PME–PME 0.14, PME–PLE 0.15. MO anterior width 0.27, posterior width 0.28, length 0.28. Clypeus 0.12 high. Leg measurements: I, 18.40 (4.78, 5.92, 6.07, 1.62); II, 13.39 (3.83, 4.32, 3.98, 1.26); III, 6.89 (2.35, 2.03, 1.87, 0.64); IV, 11.77 (4.14, 3.54, 3.34, 0.75). Chelicera: *AXu* absent; upper row with 7 teeth: *Gu* much smaller than *U2*, *U2* largest, other teeth decreasing in size gradually; *AXl* absent; lower row with 6 teeth: *Gl* largest, other teeth decreasing in size gradually. Abdomen 5.06 long, 1.7 wide, dark grey, pattern same as in male.

Epigyne (Fig. 4G, H). Fold slightly longer than wide. Vulva with 1 pair of bean-shaped spermathecae, ~ 2× longer than wide, and spaced by ~ 5× width. Central membranous sac absent.

##### Distribution.

Known only from the type locality (Fig. 7).

#### ﻿ Tetragnatha tortilis

Taxon classificationAnimaliaAraneaeTetragnathidae

Li & Liu sp. nov.

8920B59C-0B8C-58F5-A07D-B9B5D6FE6B2B

https://zoobank.org/FD801E2B-0F51-44B4-84A0-C57EF7519B93

Figs 5 , 6 , 7

##### Type material.

***Holotype*** ♂: China, Yunnan Province, Tengchong County: Jietou Township, Datang Village: 25.4277°N, 98.4129°E, 1952 m, 18.V.2006, P. Hu leg. (Hu060518). ***Paratypes***: 1♂ 1♀, same data as holotype (Hu060518); 1♀, 25.7456°N, 98.6963°E, 2030 m, 20.V.2006, X.J. Peng & P. Hu leg. (Peng060520); 1♂, 25.4202°N, 98.4095°E, 1870 m, 17.V.2006 (Peng060517), 2♂♂, 25.7456°N, 98.6963°E, 2030 m, 15.V.2006 (Peng060515), 3♂♂ 2♀♀, 25.7572°N, 98.6946°E, 2078 m, 16.V.2006 (Peng060516), 1♂, 25.4202°N, 98.4095°E, 1878 m, 19.V.2006 (Peng060519), X. J. Peng et al. leg.; 1♂, Houqiao Township: 25.3539°N, 98.2549°E, 1785 m, 28.V.2006 (Wang060528-1), 1♀, Gaoshidong Village, 25.3986°N, 98.3053°E, 2374 m, 27.V.2006 (Wang060527-2), 3♀♀, Zhaobitang Village, 25.5380°N, 98.2094°E, 2480 m, 29.V.2006 (Wang060529-1), X.P. Wang & P. Hu leg.; 2♂♂ 1♀, Mingguang Township: Zizhi Village, Cizhu River, 25.7666°N, 98.6174°E, 2120 m, 21.V.2006, C.M. Yin & J.F. Hu leg. (YHY09).

##### Etymology.

The specific epithet is derived from the Latin adjective *tortilis* (twisted), referring to the twisted distal end of conductor.

##### Diagnosis.

**Figure 5.**
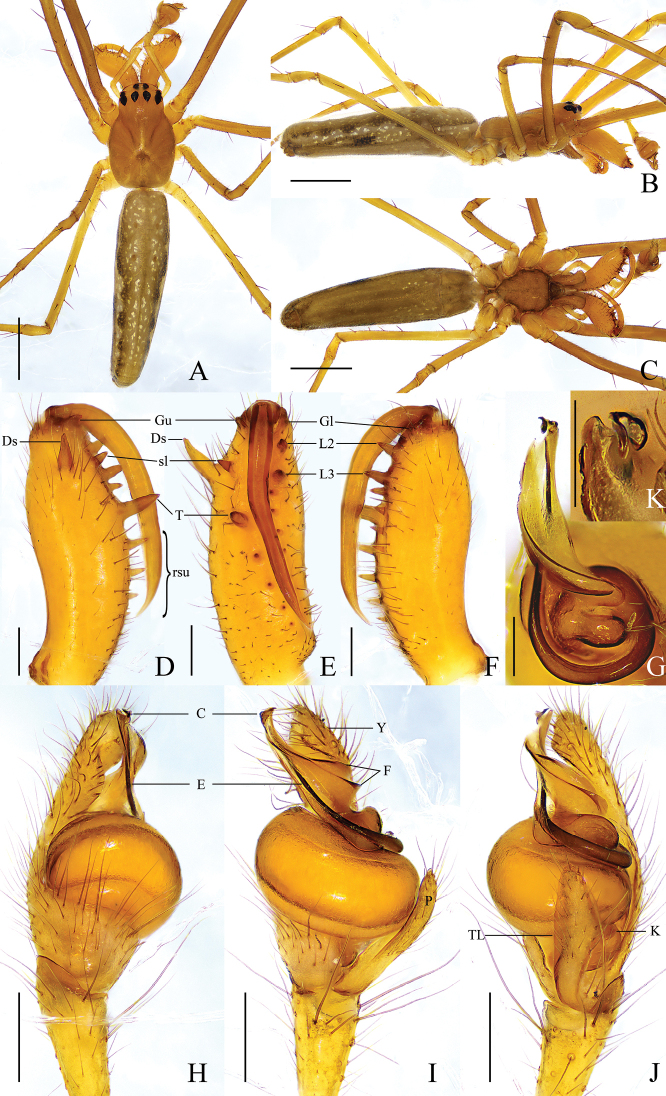
*Tetragnathatortilis* sp. nov., holotype ♂ **A–C** habitus **A** dorsal view **B** lateral view **C** ventral view **D–F** left chelicera **D** upper view **E** inner view **F** lower view **G, K** conductor and embolus detail **H–J** left palp **H** prolateral view **I** ventral view **J** retrolateral view. Scale bars: 1 mm (**A–C**); 0.2 mm (**D–F, H–J**); 0. 1 mm (**G, K**).

**Figure 6.**
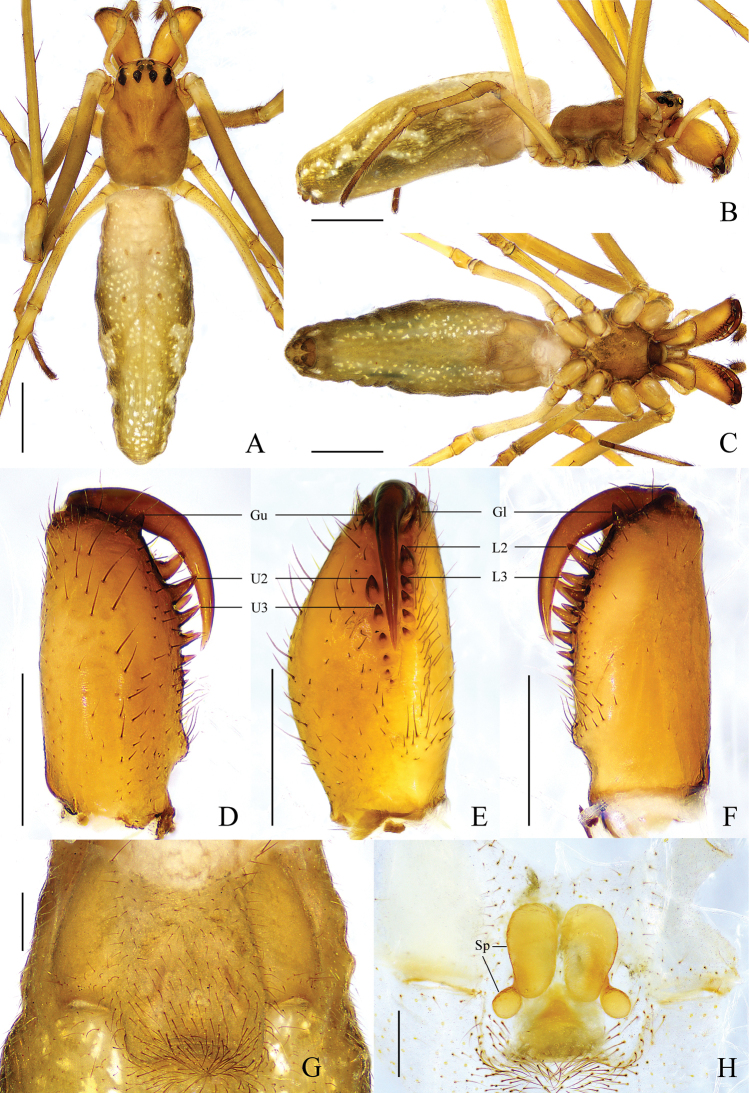
*Tetragnathatortilis* sp. nov., paratype ♀ **A–C** habitus **A** dorsal view **B** lateral view **C** ventral view **D–F** left chelicera **D** upper view **E** inner view **F** lower view **G, H** female genitalia **G** epigynal fold, ventral view **H** vulva, dorsal view. Scale bars: 1 mm (**A–C**); 0.5 mm (**D–F**); 0.2 mm (**G, H**).

**Figure 7.**
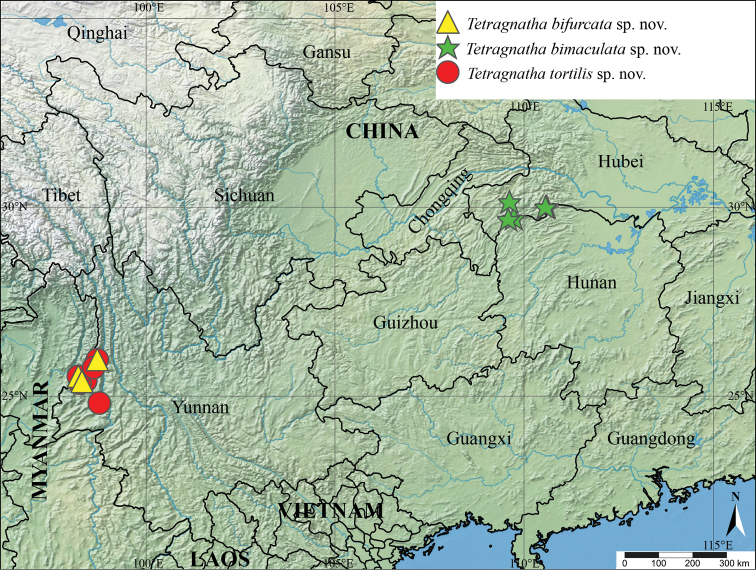
Collection localities for *Tetragnathabifurcata* sp. nov., *Tetragnathabimaculata* sp. nov., and *Tetragnathatortilis* sp. nov. in China.

The new species resembles *T.pinicola* L. Koch, 1870 (Zhu et al. 2003: figs 87A–G, 88A–G; Marusik 2010: fig. 7). Males of the two species are similar in the presence of an elongated tooth in the upper row of the chelicera in males (*T*), the conductor having 2 folds, and the shape of paracymbium, but can be distinguished by: (1) the dorsal spur of chelicera is straight in ventral view in *T.tortilis* sp. nov. (Fig. 5E) (vs. distal end curved; fig. 88C in Zhu et al. [2003]); (2) the absence of an auxiliary guide tooth of the lower row of the chelicera (Fig. 5E, F) (vs. present; fig. 88C in Zhu et al. [2003]); (3) the distal portion of conductor is twisted and has a small knot (Fig. 5G) (vs. hook-shaped; fig. 7 in Marusik [2010]). Females of the two species are similar in the presence of 2 pairs of spermathecae and the absence of a central membranous sac in the vulva, but can be distinguished by the anterior spermathecae 4× larger than posterior in *T.tortilis* sp. nov. (Fig. 6H) (vs. anterior spermathecae ~ 1/2× posterior spermathecae; fig. 87G in Zhu et al. [2003]). Both sexes of the two species can be distinguished by the sternum which is yellowish brown and without a stripe in *T.tortilis* sp. nov. (vs. dark brown with yellow stripe).

##### Description.

**Male** (holotype) (Fig. 5A–F). Total length 4.97. Carapace 1.72 long, 1.05 wide, yellowish brown, fovea, cervical, and radial grooves distinct. Eye sizes and interdistances: AME 0.06, ALE 0.07, PME 0.08, PLE 0.09; AME–AME 0.10, AME–ALE 0.14, PME–PME 0.15, PME–PLE 0.13, MO anterior width 0.24, posterior width 0.31, length 0.27. Clypeus 0.10 high. Labium brown, with thickened edge. Sternum yellowish brown with dark edge. Legs yellowish brown, with sparse spines. Leg measurements: I, 15.59 (4.04, 5.27, 4.87, 1.41); II, 9.96 (2.90, 3.10, 2.98, 0.98); III, 4.46 (1.50, 1.24, 1.12, 0.60); IV, 8.96 (2.93, 2.64, 2.66, 0.73). Chelicera: yellow, ~ 2/3 length of carapace; dorsal spur tapered; *AXu* absent; upper row with 7 teeth: *Gu* curved and almost equal to *sl* in size, *T* present, 4 *rsu* decreasing in size gradually; *AXl* absent; lower row with 7 teeth: *Gl* tiny, *L2* slightly smaller than *L3*, *L3* largest, all other teeth smaller than *L2* and almost equal in size. Abdomen 3.23 long, 0.85 wide, dorsum grayish yellow, dark folium covering almost complete dorsum, with scattered pale spots and a brown longitudinal line medially, 2 pairs of sigillae; venter grayish yellow, anterior part with sparse pale spots.

Palp (Fig. 5G–K). Paracymbium with blunt distal end, notch inconspicuous, translucent lobe elongated, extended to the end, ~ 1/3 of the width of paracymbium, knob spherical. Tegulum oval, ~ 2× wider than long. Conductor with 2 folds, distal portion twisted considerably, and directed to dorsal side. Embolus partially enveloped by conductor.

**Female** (paratype Hu060518) (Fig. 6A–F). Total length 5.70. Carapace 1.77 long, 1.24 wide. Eye sizes and interdistances: AME 0.07, ALE 007, PME 0.08, PLE 0.08; AME–AME 0.10, AME–ALE 0.18, PME–PME 0.12, PME–PLE 0.14. MO anterior width 0.25, posterior width 0.33, length 0.3. Clypeus 0.07 high. Leg measurements: I, 15.33 (4.21, 5.05, 4.73, 1.34); II, 9.48 (2.91, 2.94, 2.70, 0.93); III, 4.4 (1.48, 1.25, 1.00, 0.67); IV, 8.25 (2.72, 2.50, 2.28, 0.75). Chelicera: *AXu* absent; upper row with 7 teeth: *Gu* curved, *U2* largest, other teeth decreasing in size gradually; *AXl* absent; lower row with 6 teeth: *Gl* almost equal to *L3* in size, *L2* largest, other teeth decreasing in size gradually. Abdomen 3.92 long, 1.32 wide, dorsum pale yellow; venter with pale spots on both sides; otherwise, remaining pattern same as in male.

Epigyne (Fig. 6G, H). Fold ~ 3× wider than long. Vulva composed of 2 pairs of spermathecae, anterior pair larger, oval and almost touched each other, posterior pair smaller, spherical, and spaced by 3 diameters. Central membranous sac absent.

##### Distribution.

Known only from the type locality (Fig. 7).

## Supplementary Material

XML Treatment for Tetragnatha

XML Treatment for Tetragnatha bifurcata

XML Treatment for Tetragnatha bimaculata

XML Treatment for Tetragnatha tortilis
